# Child Feeding Practice and Primary Health Care as Major Correlates of Stunting and Underweight among 6- to 23-Month-Old Infants and Young Children in Food-Insecure Households in Ethiopia

**DOI:** 10.1093/cdn/nzaa137

**Published:** 2020-08-22

**Authors:** Zelalem Tafese, Fikadu Reta Alemayehu, Anchamo Anato, Yifru Berhan, Barbara J Stoecker

**Affiliations:** School of Nutrition, Food Science and Technology, Hawassa University, Hawassa, Ethiopia; School of Nutrition, Food Science and Technology, Hawassa University, Hawassa, Ethiopia; School of Nutrition, Food Science and Technology, Hawassa University, Hawassa, Ethiopia; St. Paul's Hospital Millennium Medical College, Addis Ababa, Ethiopia; Department of Nutritional Sciences, Oklahoma State University, Stillwater, OK, USA

**Keywords:** stunting, zinc supplements, iodized salt, diet diversity, maternal income

## Abstract

**Background:**

Child undernutrition is a major public health problem in Ethiopia. Stunting is highest in food-insecure areas and insufficient evidence may impair the design of suitable interventions.

**Objectives:**

This study aimed to identify key factors contributing to undernutrition among 6- to 23-mo-old children.

**Methods:**

A community-based cross-sectional study in food-insecure areas of Amhara and Oromia regions in April-June, 2018, enrolled 464 mother–child dyads. Bivariate and multivariate logistic regression analyses were conducted.

**Results:**

The prevalence of stunting (43.1%), wasting (12.3%), and underweight (27.3%) were high. Factors significantly associated with both stunting and underweight were child age of 12–23 mo (vs 6–11 mo), female, more siblings, lack of zinc supplement for diarrhea, inadequate diet diversity, and lack of iodized salt in complementary food.

**Conclusions:** Our findings support the need to emphasize appropriate child feeding practices and iodized salt utilization. Improvement of primary health care services related to micronutrient supplementation and family spacing also are important to address child undernutrition in the study area.

## Introduction

Child malnutrition is a continuing problem in developing countries. Globally, 144 million children <5 y of age were stunted in 2019 (1). Stunting, defined as *z* scores < −2 relative to the reference population, is the devastating result of poor nutrition in utero and in early childhood (2). According to the estimates from UNICEF, the WHO, and the World Bank, the world is still far from eliminating malnutrition; more than half of all stunted children <5 y old were reported to live in Asia and more than one-third lived in Africa (3). The Ethiopian Demographic and Health Survey (EDHS, 2016) reported the national prevalence of stunting, wasting, and underweight among under-5 children as 38%, 10%, and 24%, respectively. The same report noted that the magnitude of undernutrition in Ethiopia showed variation from region to region with the lowest prevalence of stunting (14.6%) in Addis Ababa. The regional figures for stunting, wasting, and underweight were 46.3%, 9.8%, and 28.4% in the Amhara region and 36.5%, 10.6%, and 22.5% in the Oromia region, respectively (4).

Previous studies have summarized various risk factors for childhood undernutrition, emphasizing the roles of household food security and hygienic environments in preventing chronic malnutrition (5–7). The time from 6 to 24 mo of age is a critical period for linear growth (8), and the high nutrient needs of infants and young children at this time place them at risk of undernutrition. As a preventive strategy, the WHO supports the implementation of infant and young child feeding (IYCF) guidelines (9). However, poor feeding and health care for children continue to be major determinants of undernutrition in developing countries (10).

Malnourished children have a higher risk of death from common childhood illnesses such as diarrhea, pneumonia, and malaria (11), and children in sub-Saharan Africa have a higher mortality rate from common childhood diseases than children in high-income countries (12). However, global causes of death for the majority of children <5 y of age are preventable and treatable through simple and affordable interventions (13).

Deficiencies of micronutrients such as vitamin A, iron, and zinc, also termed hidden hunger, are among the major public health problems in Ethiopia (14, 15) and these phenomena have a negative role in the growth of children (16). A key contributing factor for micronutrient deficiencies in developing countries is poor diet quality among children related to poor diversification and nutrient inadequacy in the diet (17).

Another important contributing factor for child undernutrition is household food insecurity. According to a report of the FAO in 2016, ∼10.2 million people were estimated to be food insecure in Ethiopia (18). Furthermore, a review article based on data from 2002–2014 showed that nearly 20% of people in Ethiopia required food assistance (19). This situation is linked to recurrent food shortage and famine related to the frequent occurrence of droughts (20). As 1 of the 3 underlying causes of undernutrition, household food insecurity is assumed to affect children by compromising the quantity and quality of dietary intake (21, 22).

Furthermore, inadequate health care and poor child feeding practices promote a vicious cycle of child undernutrition by contributing to the long-term existence of food insecurity. Chronic nutritional problems make children unable to develop and work to their full capacity in adult life and may be one reason that food-insecure households fail to produce enough food, even with favorable agricultural conditions (23, 24).

Despite multiple efforts, nutrition interventions often lack evidence-based information to suggest which interventions would be most successful in reducing undernutrition in specific areas. Programs and interventions may be introduced based on assumptions and unjustified conclusions, and are usually evaluated after implementation (25, 26). Inappropriate programming due to lack of evidence may lead to unanticipated and suboptimal nutrition outcomes (27). The importance of contextual evidence, particularly in a highly diverse country like Ethiopia, must be emphasized to address key contributing factors for the high magnitude of undernutrition among children.

The primary outcome measures of the present study were the anthropometrics of the 6- to 23-mo-old child assessed as length-for-age, weight-for-age, and weight-for-length *z* scores using WHO standards (28). Sociodemographic variables, as well as maternal and child health care and feeding practices, were independent variables. Knowing the contribution of these factors to undernutrition is a prerequisite to developing nutrition intervention strategies, particularly in areas where child undernutrition is an important public health problem. This knowledge can guide public health planners, policy-makers, and program implementers. Identifying the sociodemographic, dietary, primary health care, and child morbidity factors (diarrhea, respiratory and ear infections) contributing to undernutrition among infants and young children was the primary aim of the present study.

## Methods

### Study setting, design, and period

This study was conducted in 5 districts purposively selected from the 13 districts where the Development Food Security Activity (DFSA) was being implemented (**Supplemental Figure 1**). Districts for the DFSA project were chosen owing to their high levels of food insecurity. Overall, the program targeted 407,891 productive safety net program (PSNP_4_) clients. The 5 districts targeted for the current study were Meket, Lasta, and Sekota from the Amhara region and Chirro and Sirraro from the Oromia region. From the 5 districts, 21 kebeles (the smallest administrative unit in Ethiopia) were selected randomly. A community-based cross-sectional study was conducted from 25 April to 15 June, 2018, to assess the major child, maternal, and household correlates of stunting and underweight among 6- to 23-mo-old children in food-insecure households in Ethiopia.

### Sample size, population, and sampling procedure

The sample size of 493 was calculated using the single population proportion formula in Open-Epi with assumptions of 46.3% stunting in the Amhara region (4), a 97% confidence level, and a 5% nonresponse rate. The number of mother–child dyads to be selected was proportionally allocated to the kebeles in the 5 districts based on the total number of households with 6- to 23-mo-old children in each kebele. Two children who were bedridden and their mothers were not included in the study. The study participants were selected by simple random sampling from the list of food-insecure households targeted for PSNP_4_ using a random number table and 464 agreed to participate.

The sample size for this study was based on a stunting rate of 46.3% and a very conservative β of 3% so the number of participants selected was substantially larger than usually required. With the actual stunting prevalence of 43.1% and underweight prevalence of 27.3% that we measured and a β of 20%, the sample size was still sufficient to analyze individual factors for stunting and underweight, but near to the minimum for wasting. Therefore, the table showing factors associated with wasting is included as supplementary material (**Supplemental Table 1**).

### Human subjects

Ethical clearance was secured from the Institutional Review Board (IRB) of Hawassa University College of Medicine and Health Sciences. The study was also reviewed by the IRB at Oklahoma State University. Data were collected after getting permission from the district health office and obtaining informed consent from the eligible participants.

### Data collection and quality control

Data were collected using structured questionnaires (see **Supplemental Document 1**) via individual interviews. To reduce social desirability effects, particularly with respect to the individual's income, food insecurity, and care of their children, we clarified the objectives of the study, interviewed participants in privacy, and ensured the confidentiality of their information. In addition to the principal investigator (PI), 14 data collectors, 2 supervisors with a BSc degree, and 2 community health workers were involved in data collection in each kebele. Two days of intensive training on the questionnaire and data collection techniques were given by the PI. The data collectors were supervised during data collection and the collected data were checked for completeness and quality daily by the supervisors and PI. Questionnaires were pretested in 25 households in nonselected kebeles. Based on the pretest, some questions were rephrased and reordered to interview respondents smoothly, to improve clarity, and to shorten the time of administration.

### Data collection tool and measurements

The sociodemographic and the health questionnaires were adapted from the 2016 EDHS (4). Data on child morbidity were collected by asking the mother if the child had diarrhea, a respiratory infection, or an ear infection during the 2 wk before the survey. Household food insecurity was classified as recommended by the Food and Nutrition Technical Assistance guideline (29).

After removing shoes and extra clothing, child weight was measured to the nearest 0.1 kg using a calibrated SECA electronic balance with a measuring range to 25 kg. Instrument calibration was checked before weighing each child and the weighing scale was tested daily against a standard weight for accuracy. The length of 6- to 23-mo-old children was measured using a length board in a recumbent position and read to the nearest 0.1 cm (30).

The dietary diversity score (DDS) was developed from a single-pass 24-h recall by asking mothers about all foods the child had consumed for meals and snacks in the 24 h before the survey. The data collector wrote a list of the foods consumed; numbers of meals and snacks were summarized and foods consumed were subdivided into the 7 standardized food groups after completing the interview. The consumption of any amount of food from a food group was sufficient for it to be included. The 7 food groups were *1*) cereals, roots, and tubers; *2*) legumes and nuts; *3*) dairy products; *4*) flesh foods (any meat, fish, or poultry product); *5*) eggs; *6*) vitamin A–rich vegetables and fruits; and *7*) other fruits and vegetables. Consuming ≥4 of the 7 standardized food groups was labeled as adequate diversity and <4 food groups was inadequate (31).

Meal frequency of the child was determined by asking the mother how many times the child took solid, semisolid, or soft foods in the 24 h preceding the survey. Accordingly, ≥2 times for breastfed infants aged 6–8 mo, ≥3 times for breastfed children aged 9–23 mo, and 4 times for nonbreastfed children aged 6–23 mo were considered to mean the children received the minimum meal frequency (32).

### Data analysis

Data were edited, coded, and double entered into EpiData 3.1 (Epidata Association, Denmark) and analyzed using the Stata 14 (StataCorp LLC, College Station, Texas, USA) (33) statistical package. Descriptive statistics were compiled for sociodemographic variables. To describe the nutritional status of children, the Emergency Nutrition Assessment for Standardized Monitoring and Assessment of Relief and Transitions (ENA for SMART, 2011) software (Action Against Hunger, Canada and US Agency for International Development) was used to convert the weight and length for age into WHO *z* scores. Frequency, mean, and SD were calculated for continuous variables.

Collinearity was checked by the variance inflation factor and noncollinear variables were included individually in bivariate logistic regression models to assess possible associations with outcome variables. To estimate crude ORs separately for each of the anthropometric measures, factors expected to predict these nutritional outcomes were tested 1 factor at a time. Those variables with a *P* value < 0.25 in the bivariate logistic regression models were entered into the multivariable logistic regression model based on the Wald test for logistic regression (34). However, no variables with a *P* value < 0.05 in bivariate analyses were associated with a significant adjusted odds ratio (AOR) for stunting or underweight and maternal income (*P* = 0.063) was the only factor with *P* > 0.05 in bivariate analysis to be associated with a significant AOR for wasting. Multivariate logistic regressions were fitted using a stepwise backward elimination technique to identify determinants of stunting, wasting, and underweight separately and AORs with a *P* < 0.05 and their corresponding 95% CIs were computed.

## Results

### Sociodemographic characteristics

A total of 493 mother–child dyads were selected for the study; of these, 464 agreed to participate resulting in a response rate of 94%. From the dyads, 51.7% of children were females (**Table 1**). The mean ± SD age of the children was 14.6 ± 4.6 mo and the mean household size was 5.0 ± 1.8 persons. Of the households, 12.7% had ≥3 children <5 y old. Nearly 91% of the mothers were housewives and 64.7% had no formal education.

**TABLE 1:** Socioeconomic and demographic characteristics of the respondents from food-insecure households in rural communities of Amhara and Oromia regions, 2018^1^

Variables	*n*	Percentage
Age of the index child, mo		
6–11	124	26.8
12–23	338	73.2
Gender of the index child		
Male	224	48.3
Female	240	51.7
Marital status		
Not married	33	7.1
Married/living together	396	85.3
Divorced/widowed/separated	35	7.5
Educational status of mother		
Secondary school and above	23	5.0
Primary school (1–8)	140	30.3
No formal education	301	64.7
Educational status of father		
Secondary school and above	66	14.2
Primary school	164	35.3
No formal education	234	50.4
Occupation of mother		
Housewife	421	90.7
Farmer	21	4.5
Other employment	3	0.6
Merchant	16	3.4
Daily laborer	3	0.6
Occupation of father		
Farmer	137	29.5
Other employment	20	4.3
Merchant	301	64.9
Daily laborer	6	1.3
Mother had own income	316	68.1
Income of mother per month, Eth.birr		
≥500	65	14.1
<500	399	85.9
Household food security level		
Food secure	154	33.2
Mild food insecurity	78	16.8
Moderate and severe foodinsecurity*	232	50.0
Head of family		
Mother	68	14.6
Father	396	85.3
Family size		
<5	212	45.7
≥5	252	54.3
Children <5 y old, *n*		
≤2	405	87.3
>2	59	12.7

^1^ *n* = 464. 1 US$ = 27 Eth.birr. Eth.birr, Ethiopian birr; index child, child 6–23 mo old enrolled for the study.

*Only 2 households (0.43%) were severely food insecure.

### Obstetric characteristics of mothers

More than 90% of mothers had their first pregnancy within the age range of 15–26 y (**Table 2**). Nearly 27% of mothers had had ≥5 live births. A limited number (20.9%) of mothers had had ≥4 antenatal care visits for this most recent pregnancy. Only 3.5% of mothers gave birth to their most recent child at a health facility and 52.4% of mothers did not have postnatal care even once after their most recent delivery. Furthermore, 97.2% of mothers did not get a postpartum vitamin A supplement after this delivery as recommended by the WHO (35).

**TABLE 2:** Obstetric characteristics of the respondents from food-insecure households in rural communities of Amhara and Oromia regions, 2018^1^

Variables	*n*	Percentage
Age of the mother at first pregnancy, y		
<15	28	6.0
15–26	424	91.4
27–38	12	2.6
Total births, *n*		
<5	340	73.3
≥5	124	26.7
Antenatal care visits, *n*		
≥4	97	20.9
3	181	39.0
1–2	114	24.6
Not at all	72	15.5
Delivery place of the index child		
Health facility	16	3.5
Home with health extension worker	196	42.2
Home with family	252	54.3
Postnatal care visits, *n*		
≥4	3	0.6
3	124	26.7
1–2	94	20.3
Not at all	243	52.4
Birth order of the index child		
First	73	15.7
Second	112	24.1
Third	94	20.3
Fourth	70	15.1
Fifth and above	115	24.8
Mother had vitamin A supplement after birth of the index child	13	2.8
Mother had iron/folate supplement during pregnancy of the index child	276	59.5

^1^ *n* = 464. Index child, child 6–23 mo old enrolled for the study.

### Child feeding practices

Only 52.6% of mothers reported initiating complementary food for the child at 6 mo of age (**Table 3**). The meal frequency reported on the 24-h dietary information was adequate for 57.5% of the children. Overall, 21.5% of children had adequate dietary diversity. Moreover, the mean ± SD DDS of the study participants was 2.47 ± 1.34 (**Table 4**). Almost all children (94%) ate cereal staple foods (wheat, sorghum, teff, corn, or barley). Only 15% ate flesh foods (meat, fish, poultry, or liver/organ meats). Eggs were consumed by 24% and vitamin A–rich (dark green or orange) vegetables and fruits by 18%. Only 11.6% of 6- to 23-mo-old children fulfilled the minimum acceptable diet criteria (a combined indicator of adequate dietary diversity and minimum meal frequency) (Table 3). Of mothers, 55% used iodized salt with complementary food; however, 74.1% of mothers reported adding the salt during cooking.

**TABLE 3:** Child feeding practices, sanitation, and health characteristics of children from rural communities of Amhara and Oromia regions, Ethiopia, 2018^1^

Variable name	*n*	Percentage
Age of index child when complementary food started, mo		
<4	16	3.5
4–6	21	4.5
At 6	244	52.6
Beyond 6	183	39.4
Index child's first complementary food		
Cow milk	120	25.9
Formula milk	11	2.4
Cereal gruel/porridge	153	33.0
Gruel/porridge mixed	154	33.2
Family food	26	5.6
Index child met the minimum diet diversity	100	21.5
Index child met the minimum meal frequency	267	57.5
Index child met minimum acceptable diet	54	11.6
Index child consumed animal source foods		
Once in a week	233	50.2
Once in 2 wk	47	10.2
Once in a month	41	8.8
During holidays only	143	30.8
Used iodized salt for complementary food preparation	255	54.9
Iodized salt added after cooking	120	25.9
Index child had respiratory illness in the last 2 wk	194	41.8
Index child had ear infection in the last 2 wk	85	18.3
Index child had diarrhea in the last 2 wk	174	37.5
Index child had treatment from health facility for this diarrheal episode	83	47.6
Index child who got zinc among those with diarrhea in the last 2 wk	44	53.0
Index child had zinc for ≥1 prior diarrheal episode	75	16.2
Index child immunized for age	422	90.9
Index child had growth monitoring at least once	246	46.9
Index child received vitamin A supplement in the last 6 mo	328	70.7
Household used treated drinking water	93	20.1
Household had latrine	374	80.6
Mothers washed hands at all critical times	99	21.3

^1^ *n* = 464. Index child, child 6–23 mo old enrolled for the study.

**TABLE 4:** DDS of infants and young children (6–23 mo old) from food-insecure households of Amhara and Oromia regions, Ethiopia, 2018^1^

Food groups recalled by mother for previous day and night	*n*	Percentage
Starchy staples	439	94.6
Legumes and nuts	281	60.6
Dairy products (milk, yogurt, cheese)	119	25.7
Flesh foods (meat, fish, poultry, liver/organ meats)	70	15.1
Eggs	115	24.8
Vitamin A–rich (yellow, dk green, and orange colored) vegetables and fruits	85	18.3
Other fruits and vegetables	51	10.1
DDS, overall		
Mean ± SD	2.47 ± 1.34	
Min, max	1, 7	
Scored <4 food groups	364	78.45
Scored ≥4 food groups	100	21.55

^1^ *n* = 464. DDS, dietary diversity score.

### Child health characteristics

In the 2 wk preceding the survey, 42% of mothers reported their children had respiratory infections, 18% reported an ear infection, and 37% reported their children experienced diarrhea (Table 3). Treatment from a health care provider was sought for 47.6% of children with diarrhea but only 44 of these 83 children (53%) received zinc supplements. In total, 16% of mothers reported their child had had zinc supplements at least once in their life as a treatment of diarrhea.

### Prevalence of undernutrition among children

Of 6- to 23-mo-old children, 43.1% (95% CI: 37.9%, 48.7%) were stunted, 12.3% (95% CI: 9.3%, 15.3%) were wasted, and 27.3% (95% CI: 23.3%, 31.7%) were underweight (**Table 5**). The children in the 12- to 23-mo group had a higher prevalence of stunting and underweight than the 6- to 11-mo age group.

**TABLE 5:** The prevalence of stunting, wasting, and underweight among 6- to 23-mo-old children by age group in Amhara and Oromia regions, Ethiopia 2018^1^

		Stunting		Wasting		Underweight	
Age, mo	*n*	Moderate freq. (%) (LAZ < −2 to ≥3)	Severe freq. (%) (LAZ < −3)	Moderate freq. (%) (WLZ < −2 to ≥3)	Severe freq. (%) (WLZ < −3)	Moderate freq. (%) (WAZ < −2 to ≥3)	Severe freq. (%) (WAZ < −3)
6–11	126	11 (8.7)	15 (11.9)	5 (4.0)	9 (7.1)	20 (15.9)	4 (3.2)
12–23	338	92 (27.2)	82 (24.3)	24 (7.1)	19 (5.6)	72 (21.3)	31 (9.2)
Total	464	103 (22.1)	97 (20.5)	29 (6.25)	28 (6.1)	92 (19.8)	35 (7.5)

^1^ *n*  = 464. LAZ, length-for-age *z* score; WAZ, weight-for-age *z* score; WLZ, weight-for-length *z* score.

### Correlates of stunting and underweight

The multivariate logistic regression model (**Table 6**) detected associations with stunting at *P* < 0.01 for age, gender, and zinc supplements as well as associations at *P* < 0.05 for parity, iodized salt intake, and dietary diversity of the child. Among these variables, >4 times higher likelihood of stunting was found among children in the 12- to 23-mo group (AOR: 4.21; 95% CI: 2.52, 7.05) than among 6- to 11-mo infants. Girls were 1.8 times more likely to be stunted (AOR: 1.84; 95% CI: 1.23, 2.75) than were boys. Similarly, children who had never had zinc supplements were >2 times more likely to be stunted (AOR: 2.41; 95% CI: 1.33, 4.38). Likewise, 1.5 times higher likelihood of stunting was found among children whose mothers didn't utilize iodized salt (AOR: 1.55; 95% CI: 1.03, 2.32), compared with children who received iodized salt. Children whose mothers had given birth ≥5 times also were 1.7 times more likely to be stunted (AOR: 1.72; 95% CI: 1.10, 2.70) than those whose mothers had <5 births. Finally, >1.6 times higher likelihood of stunting was seen among children who consumed <4 food groups in the 24 h before the survey (AOR: 1.69; 95% CI: 1.02, 2.81) than among those who consumed ≥4 food groups. Consuming untreated water was associated with stunting in the bivariate analysis but was not significant in the multivariate model.

**TABLE 6:** Factors associated with stunting among 6- to 23-mo-old children from Amhara and Oromia regions, Ethiopia, 2018^1^

	Stunted, *n*			
Variables	Yes	No	COR (95% CI)	AOR (95% CI)
Age of index child, mo				
12–23	173	167	4.31 (2.67, 7.07)**	4.21 (2.52, 7.05)**
6–11	24	100	1	1
Gender of index child				
Female	111	113	1.72 (1.19, 2.50)**	1.84 (1.23, 2.75)**
Male	87	153	1	1
Total births, *n*				
≥5	64	60	1.63 (1.08, 2.48)*	1.72 (1.10, 2.70)*
<5	134	206	1	1
Index child supplemented with zinc for diarrhea at least once				
No	179	210	2.51 (1.43, 4.38)**	2.41 (1.33, 4.38)**
Yes	19	56	1	1
Index child's foods had iodized salt				
No	106	103	1.82 (1.25, 2.64)**	1.55 (1.03, 2.32)*
Yes	92	163	1	1
Diet diversity of index child, food groups				
<4	164	200	1.59 (1.00, 2.52)*	1.69 (1.02, 2.81)*
≥4	34	66	1	1
Treated drinking water				
No	167	204	1.63 (1.01, 2.63)*	1.17 (0.69, 1.99)
Yes	31	62	1	1

^1^ *n* = 464. AOR, adjusted odds ratio; COR, crude odds ratio; index child, child 6–23 mo old enrolled for the study. ^*,**^Significant difference from reference category: *P < 0.05; **P < 0.01.

Our model (Supplemental Table 1) detected gender, maternal income, and untreated drinking water to be associated (*P* < 0.05) with wasting. However, because of the limited number of wasted children, the power may not have been sufficient to fully evaluate other factors contributing to wasting and the model will not be discussed further.

Regarding underweight, the multivariate logistic model (**Table 7**) detected an association of gender and diet diversity with underweight at *P* < 0.01 and associations of child age, the number of births, zinc supplements, and maternal income at *P* < 0.05. Children aged 12–23 mo were 1.9 times more likely to be underweight (AOR: 1.92; 95% CI: 1.14, 3.24) than infants aged 6–11 mo. Girls were 1.8 times more likely to be underweight (AOR: 1.88; 95% CI: 1.22, 2.90) than boys. Likewise, children who had never received zinc supplements were >2 times more likely to be underweight (AOR: 2.29; 95% CI: 1.14, 4.61) than those who had taken zinc supplements at least once. Children who ate from <4 food groups had 2.3 times higher likelihood of underweight (AOR: 2.34; 95% CI: 1.27, 4.33) than those who ate from ≥4 food groups. Finally, 2.3 times higher odds of underweight were found among children whose mothers’ monthly income was <500 Ethiopian birr (Eth.birr) (AOR: 2.34; 95% CI: 1.18, 4.65) than among those children whose mothers’ monthly income was ≥500 Eth.birr.

**TABLE 7:** Factors associated with underweight among 6- to 23-mo-old children from Amhara and Oromia regions, Ethiopia, 2018^1^

	Underweight, *n*			
Variables	Yes	No	COR (95% CI)	AOR (95% CI)
Age of index child, mo				
12–23	101	237	1.77 (1.07, 2.93)*	1.92 (1.14, 3.24)*
6–11	24	101	1	1
Gender of index child				
Female	73	151	1.70 (1.12, 2.57)*	1.88 (1.22, 2.90)**
Male	53	187	1	1
Total births, *n*				
≥5	43	81	1.64 (1.05, 2.56)*	1.67 (1.05, 2.66)*
<5	83	257	1	1
Index child supplemented with zinc for diarrhea at least once				
No	114	275	2.44 (1.24, 4.79)*	2.29 (1.14, 4.61)*
Yes	12	63	1	1
Diet diversity of the index child, food groups				
<4	110	254	2.27 (1.27, 4.05)**	2.34 (1.27, 4.33)**
≥4	16	84	1	1
Maternal income per month, Eth.birr				
<500	114	277	2.09 (1.08, 4.03)*	2.34 (1.18, 4.65)*
≥500	12	61	1	1

^1^ *n* = 464. 1 US$ = 27 Eth.birr. AOR, adjusted odds ratio; COR, crude odds ratio; Eth.birr, Ethiopian birr; index child, child 6–23 mo old enrolled for the study. ^*,**^Significant difference from reference category: *P < 0.05; **P < 0.01.

## Discussion

High prevalence of stunting and underweight among children in the present study suggests severe public health significance of problems that need intervention. Our findings on the prevalence of stunting are consistent with previous studies from Uganda and Ethiopia (36, 37), but much higher than stunting rates reported from Egypt (25%) (38) and Sri Lanka (17.1%) (39). However, higher rates of stunting have been reported from the Central African Republic (61.5%) (40) and Kenya (51%) (7).

Even though an association between household food insecurity and stunting has been reported in previous studies (41, 42), our study did not show significant associations of this variable with either stunting or underweight. Perhaps a lack of statistical symmetry on the distribution of households in our study contributed because the study specifically was conducted in food-insecure areas.

Unlike several previous studies (43–45), the present study revealed girls were more stunted than boys. However, a study in food-insecure households of Pakistan (42) reported that young girls were nearly 3 times more likely to be stunted than boys. In the context of Ethiopia, where girls have historically experienced discrimination (46), more preference may have been given to the needs of boys, especially as the household experienced greater food stress. A study from Bangladesh also noted that girls were more likely to be stunted than boys in childhood, whereas boys were more likely than girls to be stunted in adolescence (47).

Consistent with previous reviews from Ethiopia (48, 49), the present study found the likelihood of being stunted to be >4-fold greater among children aged 12–23 mo than among infants aged 6–11 mo. Malnutrition becomes increasingly severe as the child's nutrient needs from complementary food increase and breast milk meets less of these needs, particularly at the age of ≥1 y (50). Also at this age, children start exploring things in their environment and are likely to put everything in their mouths, because mouthing is an important component of childhood development (51). This behavior increases the risk of repeated infection, particularly in areas with poor sanitation (52), and may result in profound effects on intestinal absorption and impair growth (53).

Children who did not meet the minimum dietary diversity criteria were at a higher risk of both stunting and underweight. Dietary diversity has been suggested as a proxy indicator of diet quality and nutrient adequacy; hence, the nutritional quality of the child's diet is improved with a diverse diet (47, 53, 54). Dietary diversification is important to meet the requirements for essential vitamins and minerals as well as protein and energy, particularly for those who are at risk of nutrient deficiencies like children in food-insecure households. Inadequate diet diversification among children was revealed to be a problem in the present study, and lower diet diversity was associated with a higher likelihood of stunting and underweight.

In this study, the consumption of animal source foods (ASFs) was not significantly associated with the anthropometric status of children. However, ASF has the potential to affect the linear growth of children, and diets poor in energy, protein, and micronutrients can be important contributing factors for stunting (55). In this study, more than two-thirds of households reported some degree of food insecurity which might be a reason only about one-quarter of children had consumed milk products or eggs the day before the survey and only 15% had consumed meat. Approximately half of the children had received any ASF less than once per week. Although small amounts of ASF can substantially increase nutrient adequacy, particularly for young children (56), consumption may have been too low in food-insecure households to have a significant impact on stunting.

The present study also revealed a higher likelihood of stunting among children who never received zinc supplements for diarrhea and whose mothers didn't utilize iodized salt for complementary foods. This result links well with the existence of deficiencies of zinc and iodine among children in developing countries (57, 58) and with the recommendations of previous studies to include zinc supplementation as one component of national strategies to reduce stunting in children (59–62).

Although there is a high prevalence of zinc deficiency among infants in Ethiopia (63, 64), poor performance on zinc supplementation has been reported by the recent EDHS (4). According to the EDHS, treatment from a health facility or provider for a child with diarrhea was sought for nearly 43% of all under-5 children but zinc supplements were given for only 33% of those who sought treatment. Another recent study also confirmed poor management and inadequate treatment among children with acute diarrhea in northern Ethiopia (65). The fact that low achievement of zinc supplementation among children during diarrhea was significantly associated with both stunting and underweight in the study area suggests a need for improvement in health care services.

The other factor associated with stunting in the present study was iodized salt utilization. Children whose mothers did not utilize iodized salt were 1.5 times more likely to be stunted. A low level of iodized salt utilization has been reported in previous studies in Ethiopia (66, 67). The association of iodized salt with the growth of children might be explained because iodine is one of the micronutrients with an important role in the secretion of growth hormone, particularly in early life, and the deficiency may result in impaired somatic growth (68).

The present study showed that low iodized salt intake may have resulted both from not using iodized salt for complementary food preparation and from poor utilization (adding iodized salt during cooking). This suggests maternal education as one strategy to improve proper iodized salt utilization with complementary food and this could be emphasized during nutrition education for mothers in the study area.

In our study, as the number of siblings increased, so too did the odds of being stunted and underweight. This finding is supported by another study from Ethiopia which stated that children whose mothers gave birth to >4 children were more likely to be stunted than children from mothers who had given birth to only 1 child (37). This may occur because families with more children may face difficulty in providing the daily nutrition requirements for proper child physical development. In our study population, 12.7% of families had >2 children <5 y old. These closely spaced pregnancies may have reduced the duration of breastfeeding for each child and thus contributed to the significant associations between the number of siblings and both stunting and underweight. In addition, as the size of a family increases, there may be a scarcity of resources for the household, especially for food and health care, which ultimately may lead to stunted growth. Furthermore, parents with many children may lack adequate time to pay proper attention to the needs of each child.

The 5 factors identified in our study that had significant associations with both stunting and underweight may be particularly important targets for intervention in the study population. A clear risk of undernutrition was associated with the increased age of the child. This finding suggests a need for initiatives focused on improving IYCF feeding practices and diet diversity, particularly those associated with complementary feeding. Failure to receive zinc supplementation for the treatment of diarrhea increased nutritional risk as did having more siblings, which suggests targets for improved implementation in the health care system. Additional investigation of an elevated risk among female children may be needed in food-insecure households.

Evidence-based nutrition intervention focusing on identified limiting factors in the early life of the child would be expected to have a positive effect on stunting reduction. Even in places with adequate resources, factors such as educational status, nutritional knowledge, and contacts with health care have important roles in the growth of children (69). A broad range of information may affect the relations between feeding practices and child growth (70, 71). The determinants of stunting are varied, and there is no single cause for undernutrition among children. Important pathways for effective interventions to mitigate stunting and promote healthy growth require clarification and consideration of context (5, 72–74). However, epidemiological studies have reported repeatedly that suboptimal breastfeeding and complementary feeding practices, recurrent infections, and micronutrient deficiencies are important proximal determinants of stunting (75, 76). The importance of a broad range of approaches to mitigate both stunting and underweight is emphasized.

The present study is one of a few studies in Ethiopia to investigate the determinants of undernutrition specifically in food-insecure settings among the most vulnerable population group: infants and young children aged 6–23 mo. Despite the possibility of recall or social desirability biases during data collection, measurement errors, and use of only 24 h of dietary information, the data revealed factors that could direct important nutrition interventions and future research initiatives.

In conclusion, child undernutrition is a severe public health concern in these food-insecure regions. The prevalences of stunting, wasting, and underweight were higher than the national averages. Accessing zinc supplementation, utilization of iodized salt, and improvement and diversification of complementary feeding practices are vital to reduce the high burden of undernutrition in the study area. Nutrition education to improve IYCF practices using the existing community-based programs, as well as enhancing the quality of primary health care to improve micronutrient supplementation and family spacing, are vital to accelerating improvement in children's nutritional status. Furthermore, increasing maternal income may contribute to addressing the high prevalence of undernutrition.

The inability to infer a causal relation of the aforementioned factors for the nutritional status of children remains one of the limitations of the present cross-sectional study, and the small number of wasted children in this sample did not allow further in-depth analysis specific to wasting. Further detailed studies are recommended to evaluate which nutrition interventions best address the challenges of undernutrition among infants and young children in food-insecure settings.

## Supplementary Material

nzaa137_Supplemental_File

## Notes

Supported by the Strengthen PSNP_4_ Institutions and Resilience-Development Food Security Activity (SPIR-DFSA) learning agenda of Hawassa University funded by the United States Agency for International Development through World Vision Ethiopia (to ZT). The contents are the responsibility of the authors and do not reflect the views of the funder. No author received travel, gifts, or honoraria as any part of this study.

Author disclosures: the authors report no conflicts of interest.

Supplemental Figure 1, Supplemental Table 1, and Supplemental Document 1 are available from the “Supplementary data” link in the online posting of the article and from the same link in the online table of contents at https://academic.oup.com/cdn/.

Abbreviations used: AOR, adjusted odds ratio; ASF, animal source food; DDS, dietary diversity score; DFSA, Development Food Security Activity; EDHS, Ethiopian Demographic and Health Survey; Eth.birr, Ethiopian birr; IRB, Institutional Review Board; IYCF, infant and young child feeding; PI, principal investigator; PSNP_4_, productive safety net program.
