# Supplementary material for: Child Feeding Practice and Primary Health Care as Major Correlates of Stunting and Underweight among 6- to 23-Month-Old Infants and Young Children in Food-Insecure Households in Ethiopia
Source: Curr Dev Nutr. 2020 Aug 22;4(9):nzaa137. doi: 10.1093/cdn/nzaa137 (PMC7475004; doi:10.1093/cdn/nzaa137)
Supplement: nzaa137_Supplemental_File [file nzaa137_supplemental_file.pdf]

Child feeding practice and primary health care as major correlates of stunting and underweight among 6-23 month infants and young children in food-insecure households in Ethiopia, Zelalem et al "Online Supplementary Material"

Supplemental Figure 1: Map of Ethiopia and the study areas in Amhara and Oromia regions, Ethiopia, 2018

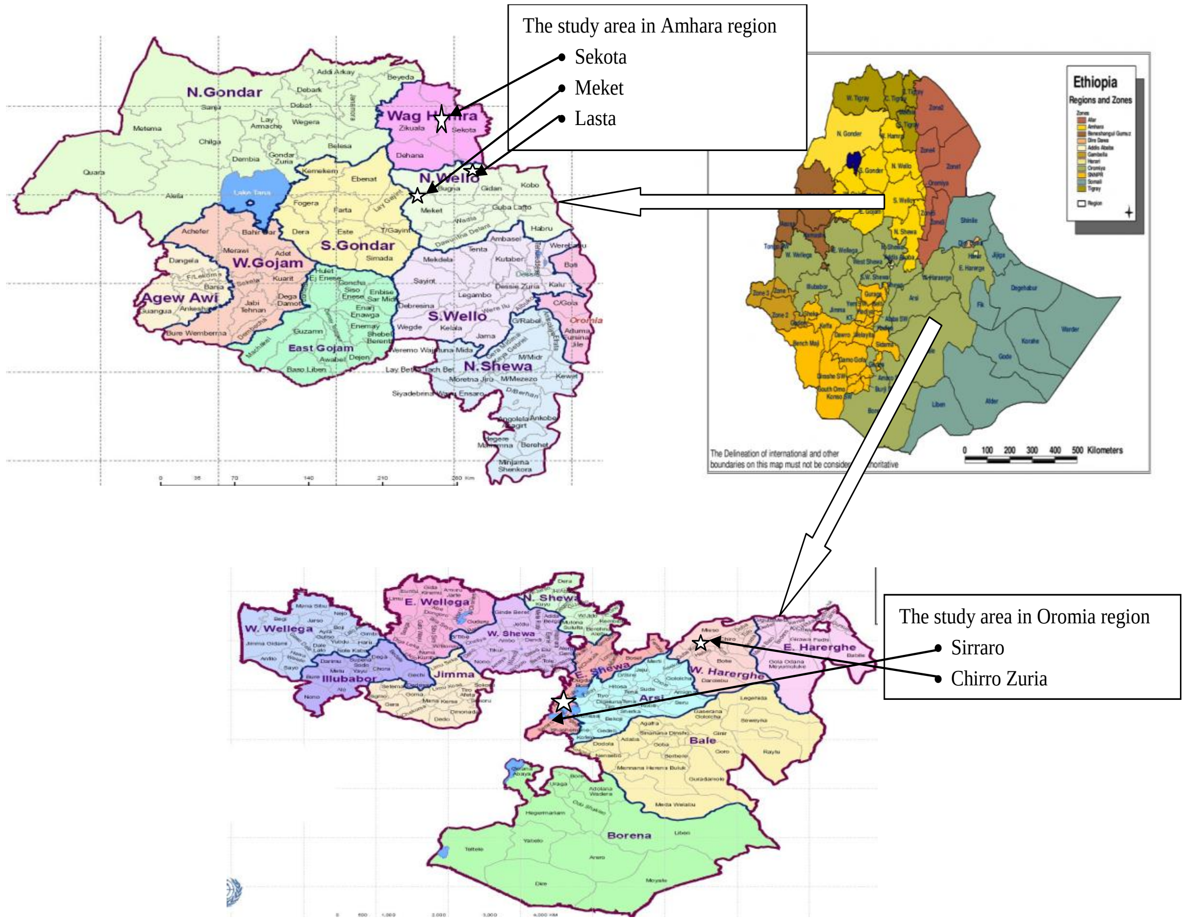

Source: http://www.ocha-eth.org /Maps/ downloadables/ Amhara. and Oromia region, accessed on 15 January 2018).

Child feeding practice and primary health care as major correlates of stunting and underweight among 6-23 month infants and young children in food-insecure households in Ethiopia, Zelalem et al "Online Supplementary Material"

Supplemental Table 1: Factors associated with wasting among 6–23 mo children from Amhara and Oromia regions, Ethiopia, 2018 (n=464)

Variables	Number wasted		COR [95% CI]	AOR [95% CI]
	Yes	No		
**Gender**				
Female	35	189	1.83 [1.04,3.23] *	1.97 [1.10,3.51] *
Male	22	218	1	1
**Received growth monitoring at least once**				
Yes	23	223	0.55 [0.31,0.98] *	0.60 [0.34,1.08]
No	34	184	1	1
**Family size**				
≥5	38	214	1.80 [1.00,3.23] *	1.70 [0.94,3.08]
<5	19	193	1	1
**Maternal income per month**				
<500Eth.birr	53	338	2.73 [0.94,7.71]	3.29 [1.14, 9.50] *
≥500Eth.birr	4	69	1	1
**Treated drinking water**				
No	52	319	2.86 [1.11,7.40] *	3.22 [1.24, 8.39] *
Yes	5	88	1	1

*Significant at P-value of <0.05

Child feeding practice and primary health care as major correlates of stunting and underweight among 6-23 month infants and young children in food-insecure households in Ethiopia, Zelalem et al “Online Supplementary Material”

Supplemental document 1: English version questionnaire, for the study in Amhara and Oromia regions, Ethiopia, 2018

QUESTIONNAIRE ENGLISH VERSION, HAWASSA UNIVERSITY

College of Agriculture, School of Nutrition, Food Science and Technology (SNFST)

Questionnaire prepared for the community-based cross-sectional study to assess the prevalence and factors associated with undernutrition among children 6-23 months of age in food insecure households of Ethiopia

Introduction and Consent

Hello. My name is ____ I am visiting you on behalf of Zelalem Tafese, PhD student of Hawassa University’s School of Nutrition, Food Science and Technology. He is conducting research for the partial fulfillment of requirements for the degree of Doctor of Philosophy in Human Nutrition. Your household is selected randomly to participate in this study and there are other households to be selected randomly in the woreda. Therefore, I am going to ask you several questions about those factors and related issues about the research. He has received permission from Hawassa University School of Medicine and Health Sciences, the Woreda council office and respective district health offices to conduct this study.

I would very much appreciate your participation in this interview. This information will help all stakeholders working on food insecurity. We assure that the interview process will not bring any harm to you and your family. Whatever information you provide will be kept strictly confidential and will not be shared with anyone other than the investigator.

Participation in this survey is voluntary, and if we should come to any question you don't want to answer, just let me know and I will go on to the next question; or you can stop the interview at any time. However, we hope you will participate in the survey since your views are important. Therefore, I politely request your cooperation to participate in this interview.

In case you want to contact the investigator, please contact, Zelalem Tafese, 0911479506

Would you be willing to participate? Yes ☐ No ☐

Signature of the respondent: ----------------

Date: --

Interviewer name: ---------------------------

Signature: -----------------------------------

Child feeding practice and primary health care as major correlates of stunting and underweight among 6-23 month infants and young children in food-insecure households in Ethiopia, Zelalem et al “Online Supplementary Material”

Date: ---

❖ Note to the interviewer on approaching for interview

- Once you get to the household, you should get permission to enter into the house. Do not rush into the house without the permission of the household members.
- Introduce yourself by name, and explain the purpose of your visit.
- Tell the household member “WHY” and “HOW” the household is selected for the study.
- Ask whether the mother/caretaker of the child is present.
- Briefly explain the purpose of the study by reading what is written on the questionnaire, and ask for her consent.
- If the mother/caretaker is not around, take an appointment for another visit.
- At the end of the interview, tell the respondent that it is the end of the interview, and thank the individual for giving her view.

THANK YOU FOR YOUR COOPERATION!!!

Date: -------------------------------

ID no: ---------------------------

Region------------Woreda-----------------------Name of cluster/kebele----------------------

PART ONE: DEMOGRAPHIC AND SOCIOECONOMIC CHARACTERISTICS

No	Questions	Response
101.	Number of household members	
102.	Number of under-five children in the household	1. ≤6 months-----------<br>2. 6-23 months----------<br>3. ≥ 23 months----------
103.	Current status of the mother	1. Pregnant<br>2. Lactating<br>99. Other
104.	Age of the mother/caretaker	--------------years
105.	Age of first pregnancy	--------------years
106.	Parity(total no. of births)	---------------
107.	Marital status of the mother/caretaker	1. Never married<br>2. Married or living together<br>3. Divorced/separated<br>4. Widowed

108.	Religion of parents/caretaker	1. Orthodox<br>2. Catholic<br>3. Protestant<br>4. Muslim<br>5. Traditional<br>99. Other
109.	Educational status of the mother/caretaker	1. Cannot read and write<br>2. Primary school(1-8)<br>3. Secondary school (9-12)<br>4. Post secondary school<br>99. Other (specify)
110.	Occupation of the mother/caretaker	1. Government employee<br>2. Housewife<br>3. Merchant<br>4. Farmer<br>5. Day laborer<br>99. Other (specify)
111.	Age of the child's father	------years
112.	Educational level of the father	1. Cannot read and write<br>2. Primary school (1-8)<br>3. Secondary school (9-prep.)<br>4. More than secondary<br>5. Other (specify)
113.	Occupation of the father	1. Government employee<br>2. Merchant<br>3. Farmer<br>4. Day laborer<br>99. Other (specify)
**Information on the wealth of the family**		
114.	Do you have your own income?	1. Yes 2. No
115.	If Yes, how much do you earn in a typical month?	__________Birr
116.	In total how much is the monthly income of your household?<br>(Data collector: Please help her to remember different sources of income)	___________Birr
117.	Does your household have a land (owned or rented) that can be used for agriculture?	1. Yes 2. No
118.	How many (Timad) of agricultural land does the household own?	1. ______units/Timad<br>98. I don't know/Not sure
119.	How many of the following animals does your household own?	Poultry -------------<br>Milk cows, oxen or bulls --------<br>Horses, donkeys, or mules -------<br>Camels -------------<br>Goats -------------<br>Sheep -------------<br>Chickens ------------<br>Beehives ---------------
120.	Does any member of this household have a bank or microfinance saving account?	1. Yes<br>2. No

121.	Type of floor material of the living house (observation)	1. Mud<br>2. Wood<br>3. Cement<br>99. Other (specify)
**Does any member of your household own**		
122.	A bed?	1. Yes 2. No
	A chair?	1. Yes 2. No
	A kerosene/pressure lamp?	1. Yes 2. No
	A table?	1. Yes 2. No
	A radio?	1. Yes 2. No
	A mobile phone?	1. Yes 2. No
	An electric Stove to bake Injera (*mitad*)?	1. Yes 2. No
	A bicycle?	1. Yes 2. No
	A fixed line telephone?	1. Yes 2. No
	An animal-drawn cart?	1. Yes 2. No
	A motorcycle?	1. Yes 2. No
	A television?	1. Yes 2. No
	A mill?	1. Yes 2. No
123.	Does your household have electricity?	1. Yes 2. No
124.	Does your house have windows?	1. Yes 2. No
125.	What is the main material of the roof your house?	1. Thatched/Leaf<br>2. Plastic sheet<br>3. Corrugated iron<br>99. If other specify ___________
126.	What is the main material of the floor of your house?	1. Natural floor<br>2. Wood or bamboo floor<br>3. Finished floor<br>99. If other specify ___________
127.	What is the main material of the walls of your house?	1. Bamboo/Wood<br>2. Stone with mud<br>3. Sticks with mud<br>4. Bricks/Cement<br>99.If other specify ___________
128.	Number of rooms in the house	---------------
129.	Source of water for drinking?	1. Pipe<br>2. Protected well/spring<br>3. Unprotected well/spring/pond<br>4. River<br>99. Other (specify)
130.	Distance of water source from residence	1. In the compound<br>2. < 30 minutes<br>3. > 30 minutes<br>4. Don't know

131.	Who **usually** collects drinking water in your family:	1. Adult woman<br>2. Adult man<br>3. Female child under 15 years old<br>4. Male child under 15 years old<br>99. other
132.	Is the water available all year round	1. Yes<br>2. No

PART TWO: QUESTIONNAIRES TO ASS'S BEHAVIORAL CONDITIONS OF RESPONDENT MOTHERS

**1.1. Child feeding practice and health seeking behavior**		
201.	Do you have a child of 6-23 months	1. Yes<br>2. No …..skip to question 203
202.	Relation of the respondent to the child	1. Mother<br>2. Caretaker
203.	Have you ever breastfed your child?	1. Yes<br>2. No ..skip to question 204
204.	What was your breast feeding practice from 0-6 months	1. Exclusive breast feeding<br>2. Partial breast feeding<br>3. Not breast feeding
205.	How many times did you breastfeed during 0-6 months within a 24 hr period.	1. <8 times<br>2. ≥ 8 times<br>3. On demand<br>98. I don't know
206.	Did you give your child colostrum?<br>(A yellow milk from mothers breast `Enger` in Amharic)	1. Yes 2. No
207.	For how long did you breastfed your last child?	----------months
208.	At what age did the child start complementary feeding?	-----------months
209.	What common food/fluid did you use for the child as a complementary food? (more than one response is possible)	1. Cow's milk<br>2. Formula milk<br>3. Gruel<br>4. Adult food<br>99. Other (specify)
210.	How often do you give your child animal source foods?(Ex. eggs, meat, fish, chicken and milk)	1. Every day<br>2. ≥ 4 times a week<br>3. Once a week<br>4. Once to several times a month<br>5. Less than once a month<br>6. Never<br>99. Other
211.	Do you separately prepare food using separate utensils for your child?	1. Yes 2. No
212.	What do you use to feed your child?	1. Hand<br>2. Cup and spoon<br>3. Cup<br>4. Bottle<br>99. Other

213.	What is the most important source of knowledge and practice to feed your child?(	1. Your family<br>2. Friends<br>3. Mass media<br>4. Health care workers<br>99. Other, specify
214.	Who is responsible to care and feed children in your family?(Circle all that the mother responds)	1. Only the mother<br>2. Husband<br>3. Female child under 15 years old<br>4. Male child under 15 years old<br>99. Other…specify,
215.	Do you think your husband should be involved in nutritional care of you and your family?	1. Yes<br>2. No
216.	Do you think male individuals in your community are actively involved in caring and feeding a child?	1. Yes<br>2. No
217.	How do you describe the involvement of your husband in caring and feeding your child? (record all mentioned?)	1. Produce food items<br>2. Giving you money to buy food items<br>3. Buy and bring food items for the child<br>4. Appreciate and encourage you while caring for the child<br>5. Help you with cooking<br>99. Other, specify
218.	What do you suggest is the reason why males are not usually involved in caring and feeding children in your community?	1. Cultural barrier<br>2. Short of time<br>3. Negligence<br>99. Other(specify)
**1.2. Questionnaire on adherence to vitamin A, zinc and iodized salt utilization**		
219.	Is your child vaccinated for his age?	1. Yes<br>A. by the response of the respondent<br>B. by checking immunization card<br>2. No
220.	Is your child updated for Vit. A supplementation? (check from the vaccination card)	1. Yes<br>A. by the response of the respondent<br>B. by checking immunization card<br>2. No
221.	Has your child had measles within the last 3 months?	1. Yes 2. No….skip to question 223
222.	If yeswas he/she treated with Vitamin A?	1. Yes 2. No
223.	How many episodes of diarrhea has the child had during the last three months?	----------------
224.	How many days did the child have diarrhea? (The most recent diarrhea)	-----------------

225.	Did you give ORS when your child had diarrhea?<br><br>("Oral rehydration salt" or "LemLem".)	1. Yes 2. No
226.	Have you ever heard about zinc?	1. Yes 2. No..skip to next
227.	Where did you hear about zinc? (more than one answer possible)	1. Radio<br>2. Television<br>3. Health care provider<br>4. Health Extension worker<br>5. Friend / Neighbors
228.	What was given to treat the diarrhea? (more than one response possible)	1. Pill or syrup Antibiotic<br>2. Anti- motility medicine<br>3. Zinc + ORS<br>4. ORS only<br>5. Unknown pill or syrup<br>6. Antibiotic injection<br>7. Non antibiotic injection<br>8. Unknown injection<br>9. Intravenous therapy
229.	Do you give ORS solution along with a zinc product for your child during the most recent diarrheal episode?	1. Yes<br>2. No<br>99. Don't know/remember
230.	How do you feed the Zinc tablet?	1. Dilute with water<br>2. Dilute with ORS<br>3. Mix with breast milk<br>99.Other..specify
231.	If you received zinc, for how many days did you give zinc for your child? (circle what the mother mentioned)	1. For 10 days<br>2. 7-10 days<br>3. 3-6 days<br>4. 1-3 days<br>5. Until the diarrhea stops<br>99. Don't know/remember
232.	If you did not give the full dose of zinc, what was the reason?	1. Unpleasant taste<br>2. The child not to take<br>3. Vomiting<br>4. The diarrhea stopped<br>98. Don't know
233.	What the reason some mothers did not use zinc when their children had diarrhea?	1. Did not know where to obtain zinc<br>2. It is not prescribed from clinic<br>3. Unsure about administering zinc<br>4. Expensive<br>99.Other
234.	Do you use iodized salt with complementary food for your child?(observe and if packed read label)	1. Yes 2. No..skip to question 237

235.	If yes, why do you choose iodized salt?	1. For health<br>2. Taste<br>3. I don't know<br>99. Others, Specify-------------
236.	If no, why don't you choose iodized salt?(circle all what the mother mentioned)	1. It has no salty taste<br>2. Not available in the market<br>3. Too costly<br>4. Finished for a while<br>5. Other, Specify………………………<br>……………………………………
237.	How often do you usually buy salt?	1. Weekly<br>2. Monthly<br>3. Other, Specify________
238.	When do you usually add salt to your food?	1. Before cooking<br>2. During cooking<br>3. After cooking<br>4. Any time
**1.3. Questionnaire on health seeking behavior of mothers**		
239.	Do you have information about a vaccination that prevents your child from diarrhea?	1. Yes 2. No
240.	Did your child receive Roto virus vaccination?	1. Yes<br>A. by the response of the respondent<br>B. by checking immunization card<br>2. No
241.	Did you receive any antenatal care during your last pregnancy?	1. Yes 2. No…skip to question 243
242.	Why do you take ANC/what initiates you to visit health institutions?	1.Health problem<br>2.Past experience<br>3.Because it is helpful for your health<br>99.Other …specify
242.	During your last pregnancy, how many months pregnant were you the first time you went to a health facility?	-------------
243.	During your last pregnancy, did you take any iron and folic acid supplements?	1. Yes 2. No
244.	During your last pregnancy, did you receive any counseling or information about nutrition for pregnant [illegible]	1. Yes 2. No
245,	During your last pregnancy, did you receive any counseling about breastfeeding?	1. Yes 2. No

246.	How many times did you get ANC during your last pregnancy?	1. 1<br>2. 2<br>3. 3<br>4. ≥4
247.	For which condition did you visit the health institutions? (more than one response possible)	1. Only during critical illness<br>2. ANC<br>3. Family planning<br>4. Delivery<br>5. Nutritional advice<br>6. Growth monitoring<br>99.Other,specify
248.	Do you think that women should have ANC when they are pregnant even though they are not sick?	1. Yes 2. No
249.	Who took the decision on seeking health care for Rx/ ANC/ determining place of delivery and the like in your family? (more than one response possible)	1. Respondent<br>2. Husband<br>3. Parent<br>4. Religious leader<br>99. other(specify)
250.	Where didyou give birth for your last child?	1. Home with family members<br>2. Home with TBA or HEW<br>3. Health institution<br>99. Other, specify
251.	After your last birth did you get PNC service?<br><br>**For the data collector:** PNC is any visit after discharge to home (within 1 week, preferably on day 3,7-14 days after birth and 4-6 weeks after birth)	1. Yes 2. No..skip to the next question
252.	How many times did you receive PNC?	1. ______times<br><br>98. I don't remember
253	During your PNC visit did you get any counseling about (More than one response possible)	1. Breast feeding<br>2. Child feeding<br>3. Weight measurement of the child<br>4. Family planning<br>99. Other specify,--------------
254.	Where did you give birth for your last child?	1. Own home<br>2. Government hospital/health centre<br>3. Private hospital<br>4. Private clinic<br>99. Other specify_______

255.	How long after a sickness beginsdo you usually seek health care for yourself or your child?	1. Immediately<br>2. The next day<br>3. After 2 days<br>4. Between 3 days and a week<br>5. After a week or more<br>99.Other (specify)------------
256.	How many times was your child sick within the last month?	1. One times<br>2. Two times<br>3. Three or more times
257.	Did you seek medical care outside your home?	1. Yes 2. No
258.	If you do not seek care outside your home, what was the reason? (circle what the mother mentioned)	1. Clinic too far from house<br>2. Unable to find transport<br>3. Cost for travel too high<br>4. Cost for treatment too high<br>5. Other children at home who could not be left alone<br>99. Other(specify)------------------
259.	If yes; where did you seek care?	1. Private clinic in your community<br>2. Government/public clinic in your community<br>3. Pharmacy<br>4. Traditional healer<br>99. Other (specify)----------
260.	If your child experiences diarrhea when will you bring him/her to the health facility from the onset of diarrhea?	1. within one day of onset<br>2. less than one day of onset<br>3. greater than one day of onset<br>99. Other specify……
261.	During illness what specific signs urge you to bring your child to the health facility?<br>(circle all the mother mentions)	1. vomiting<br>2. fever<br>3. lethargy(sleepy)<br>4. unable to drink<br>99. Other specify
262.	From the following liquid types, which one is more helpful to feed your child during the diarrheal episode?	1. Cow Milk<br>2. Breast feed<br>3. Soft drinks<br>4. ORS with zinc<br>5. Juice<br>6. Plain water<br>99.Other (specify)----------
263.	During a diarrheal episode which one is helpful for your child?	1. Increasing fluid<br>2. Decreasing fluid<br>3. Not giving fluid<br>4. Giving dry food

264.	When you compare the amount of food and fluid you give for your child during the illness with the healthy situation, it is:	1. Less than usual<br>2. Greater<br>3. Similar<br>4. Nothing given
265.	What are the main factors that hinder mothers seeking health care for their sick child? (multiple responses possible)	1. Religious factor<br>2. Shortage of time<br>3. Shortage of money<br>4. Road is not available<br>5. Do not know health care unit<br>99. Others, specify____________

1.3. Sanitation and hygiene behavior of women

266.	Did you wash your hands yesterday?	1. Yes 2. No skip to next question
267.	In which situations did you wash your hands? (Record all the mother mentioned)	1. Before food preparation<br>2. Before eating<br>3. After eating<br>4. After visiting latrine<br>5. After cleaning of child's bottom<br>6. After touching animal feces<br>99. Other (specify)-----------
268.	What did you use to wash your hands?	1. Soap & water<br>2. Ash & water<br>3. Only water<br>99. Others (specify)------------
269.	Do you have hand washing facility near to the latrine? (observation)	1. Yes<br>2. No
270	Do you have a latrine	1. Yes 2. No
271.	Ownership of the latrine	1. Private<br>2. Shared with neibours
272.	Type of latrine facility (observation)	1. Flush/pour flush to piped sewer system<br>2. Flush/pour flush to septic tank<br>3. Flush/pour flush to pit latrine<br>4. Ventilated improved pit (VIP) latrine<br>5. Pit latrine with slab<br>6. Pit latrine without slab<br>99. Other (specify)--------------------
273.	Latrine maintenance(observation)	1. No need 2. Needed
274.	If the household has no latrine, where do you dispose human waste?	1. Open field<br>99. Other (specify)----------------
275.	How do you dispose refuse?	1. Pit<br>2. Open field<br>3. Burning<br>4. Garbage can<br>99. Other

276.	What is the source of water for drinking?	1. Pipe<br>2. Protected well/spring<br>3. Unprotected well/spring<br>4. River<br>99. Other (specify)---------------------
277.	Distance from house to water source(round trip)	---------Minutes
278.	Is water for household consumption available all year round?	1. Yes 2.No
279.	Type of water collection container	1. Pot<br>2. Plastic bucket (``Yeplastic Baldi``)<br>3. Iron bucket(``Yebiret Baldi``)<br>4. Jerry can<br>99. Other
280.	How did you transport the drinking water to the house yesterday?	1. In a covered container<br>2. In an uncovered container<br>99. Other (specify
281.	How many times did you collect water for drinking yesterday?	Number---------------
282.	Do you treat your water in any way to make it safer to drink?	1. Yes<br>2. No --- skip to the next question
283.	What do you usually do to the water to make it safer to drink?	1. Boil<br>2. Bleach/chlorine added<br>3. Strained through cloth<br>4. Ceramic, sand, composite, or other filter<br>5. Solar disinfection<br>6. Let it stand and settle<br>7. No treatment<br>99. Other

PART THREE:INFORMATION OF THE INDEX CHILD

NO	Questioner	Response
301.	Age of the child	---------
302.	Sex	---------
303.	Birth order of the child	
304.	Weight	---------k.g
305.	Height	---------- c.m
306.	How many times was your child sick within the last month?	1. One times<br>2. Two times<br>3. Three or more times
307.	Did your child experience diarrhea in the last two weeks?	1. Yes<br>2. No….skip to the next question
308.	If yes how many times?	1. One times<br>2. Two times

		3. Three or more times
309.	Did your child experience respiratory tract infections (cough) in the last two weeks?	1. Yes<br>2. No….skip to the next question
310.	Did your child experience fever in the last two weeks?	1. Yes<br>2. No….skip to the next question
311.	If yes how many times?	1. One times<br>2. Two times<br>3. Three or more times
312.	Did your child experience ear problems (infection) in the last two weeks?	1. Yes<br>2. No….skip to the next question
313.	If yes how many times?	1. One times<br>2. Two times<br>3. Three or more times
314.	Did your child ever experience measles?	1. Yes 2. No
	In the past 3 months, was the weight, height or mid-upper arm circumference of your child measured?	1. Yes 2. No…skip to the next question
315.	If yes, were you given any advice or information about feeding your child after he/she was measured?	1. Yes 2. No
316.	Was your youngest??child ever treated for SAM?	1. Yes<br>2. No….skip to the next question
317.	How many times?	1. One times<br>2. Two times<br>3. Three or more times
318.	Did you receive any specific food or milk for your child as a treatment for severe acute malnutrition?	1. Infant formula<br>2. Plumpy Nut<br>3. F-100<br>99.Other …specify
319.	Was any one of your children ever treated for SAM?	1. Yes 2. No

PART FOUR: DIETARY PRACTICE OF WOMEN AND CHILDREN

1.1. Minimum Dietary Diversity for Women (MDD-W) and Meal frequency

Instruction for the interviewer: Ask the mother about foods and drinks that she ate or drank yesterday during the day or night, whether at home or anywhere else. Please help on recalling foods she may have eaten while preparing meals or preparing food for others. Tell her not to include any food used in a small amount for seasoning or condiments (like chilies, spices, herbs or fish powder). Then write the list of foods on the back of this form and code it later.

Was yesterday an unusual or special day (Festival, Funeral, fasting…) 1. YES 2. NO.

No	10 food groups in the MDD-W	Groups/rows on the MDD-W questionnaire	Response
1	Grains, white roots and tubers, and plantains	Foods made from grains<br>White roots and tubers and plantains	1. Yes<br>2. No
2	Pulses (beans, peas and lentils)	Pulses (beans, peas and lentils)	1. Yes<br>2. No
3	Nuts and seeds	Nuts and seeds	1. Yes<br>2. No
4	Dairy	Milk and milk products	1. Yes<br>2. No
5	Meat, poultry and fish	Organ meat<br>Meat and poultry<br>Fish and seafood	1. Yes<br>2. No
6	Eggs	Eggs	1. Yes<br>2. No
7	Dark green leafy vegetables	Dark green leafy vegetables	1. Yes<br>2. No
8	Other vitamin A-rich fruits and vegetables	Vitamin A-rich vegetables, roots and tubers ,Vitamin A-rich fruits	1. Yes<br>2. No
9	Other vegetables	Other vegetables	1. Yes<br>2. No
10	Other fruits	Other fruits	1. Yes<br>2. No

4.2. MINIMUMUM DIETARY DIVERSITY QUESTIONNAIRES FOR CHILDREN 6-23 MONTHS

Interviewer instruction: Ask the mother about foods and drinks that her child had yesterday during the day or night, including (breakfast/lunch/ dinner/ snacks). Ask her freely and help to recall the type of food items she fed to her child within the last 24 h. Remind her to mention whether the foods were combined or not and foods and drinks she gave as snacks as well as during any main meals. Then write the list of food that the mother mentioned on the back of this form and code it later.

No	7 food groups in children MDD	Groups/rows on the MDD questionnaire	Response
1	Grains, roots and tubers	Foods made from grains,white roots and tubers and plantains	1. Yes<br>2. No
2	Legumes and nuts	Pulses (beans, peas and lentils) ,<br>Nuts and seeds	1. Yes<br>2. No
3	Dairy products	Milk and milk products	1. Yes<br>2. No
4	Flesh foods	Organ meat<br>Meat and poultry	1. Yes<br>2. No

		Fish and seafood	
5	Eggs	Eggs	1. Yes<br>2. No
6	Vitamin A-rich vegetables and fruits	Dark green leafy vegetables Other vitamin A-rich fruits and vegetables	1. Yes<br>2. No
7	Other fruits and Vegetables	Other fruits and other vegetables	1. Yes<br>2. No

4.3 MINIMUMUM MEAL FREQUENCY QUESTIONNAIRES FOR CHILDREN 6-23 MONTHS

Interviewer instruction: Ask the mother about the number of meals the child had in the last 24 hr of the survey, during the day or night, and circle the responses of the mother.

No.	Meal	Response
1	Breakfast	0.Yes 1.No
2	Snack	0.Yes 1.No
3	Lunch	0.Yes 1.No
4	Snack	0.Yes 1.No
5	Dinner	0.Yes 1.No
6	Snack	0.Yes 1.No
Total number of meal in the last 24 hr ____________		

PART FIVE

Information Related to Household Food Security		
**Code**	**Questions**	**Remark**
1	In the past four weeks, did you worry that your household would not have enough food?	1. Yes<br>2. No → skip to 3<br>98. don't remember
2	How often did this happen?	1. Rarely (once or twice in the past four weeks)<br>2. Sometimes (three to ten times in the past four weeks)<br>3. Often (more than ten times in the past four weeks)
3	In the past four weeks, were you or any household member not able to eat the kinds of foods you preferred because of a lack of resources?	1. Yes<br>2. No → skip to 5<br>98. Don't Remember
4	How often did this happen?	1. Rarely (once or twice in the past four weeks)

		2. Sometimes (three to ten times in the past four weeks)<br>3. Often (more than ten times in the past four weeks)
5	In the past four weeks, did you or any household member have to eat a limited variety of foods due to a lack of resources?	1. Yes<br>2. No → skip to 7<br>98. don't remember
6	How often did this happen?	1. Rarely (once or twice in the past four weeks)<br>2. Sometimes (three to ten times in the past four weeks)<br>3. Often (more than ten times in the past four weeks)
7	In the past four weeks, did you or any household member have to eat some foods that you really did not want to eat because of a lack of resources to obtain other types of food?	1. Yes<br>2. No → skip to 9<br>98. Don't Remember
8	How often did this happen?	1. Rarely (once or twice in the past four weeks)<br>2. Sometimes (three to ten times in the past four weeks)<br>3. Often (more than ten times in the past four weeks)
9	In the past four weeks, did you or any household member have to eat a smaller meal than you felt you needed because there was not enough food?	1. Yes<br>2. No → skip to 11<br>3. 98. Don't Remember
10	How often did this happen?	1. Rarely (once or twice in the past four weeks)<br>2. Sometimes (three to ten times in the past four weeks)<br>3. Often (more than ten times in the past four weeks)
11	In the past four weeks, did you or any other household member have to eat fewer meals in a day because there was not enough food?	1. Yes<br>2. No → skip to 13<br>98. Don't Remember
12	How often did this happen?	1. Rarely (once or twice in the past four weeks)<br>2. Sometimes (three to ten times in the past four weeks)<br>3. Often (more than ten times in the past four weeks)

13	In the past four weeks, was there ever no food to eat of any kind in your household because of lack of resources to get food?	1. Yes<br>2. No → 15<br>98. Don't Remember
14	How often did this happen?	1. Rarely (once or twice in the past four weeks)<br>2. Sometimes (three to ten times in the past four weeks)<br>3. Often (more than ten times in the past four weeks)
15	In the past four weeks, did you or any household member go to sleep at night hungry because there was not enough food?	1. Yes<br>2. No →skip to 17<br>98. Don't Remember
16	How often did this happen?	1. Rarely (once or twice in the past four weeks)<br>2. Sometimes (three to ten times in the past four weeks)<br>3. Often (more than ten times in the past four weeks)
17	In the past four weeks, did you or any household member go a whole day and night without eating anything because there was not enough food?	1. Yes<br>2. No<br>98. Don't Remember
	How often did this happen?	1. Rarely (once or twice in the past four weeks)<br>2. Sometimes (three to ten times in the past four weeks)<br>3. Often (more than ten times in the past four weeks)
